# Investigating the association of the effect of genetically proxied PCSK9i with mood disorders using cis-pQTLs: A drug-target Mendelian randomization study

**DOI:** 10.1371/journal.pone.0310396

**Published:** 2024-09-26

**Authors:** Alisha Aman, Eric A. W. Slob, Joey Ward, Naveed Sattar, Rona J. Strawbridge

**Affiliations:** 1 The Graduate School, College of Medical, Veterinary, and Life Sciences, University of Glasgow, Glasgow, United Kingdom; 2 School of Health and Wellbeing, University of Glasgow, Glasgow, United Kingdom; 3 Department of Psychology, Education, and Child Studies, Erasmus University Rotterdam, Rotterdam, The Netherlands; 4 Department of Applied Economics, Erasmus School of Economics, Erasmus University Rotterdam, Rotterdam, The Netherlands; 5 Erasmus University Rotterdam Institute for Behaviour and Biology, Erasmus School of Economics, Rotterdam, The Netherlands; 6 School of Cardiovascular and Metabolic Sciences, University of Glasgow, Glasgow, United Kingdom; 7 Cardiovascular Medicine Unit, Department of Medicine Solna, Karolinska Institute, Stockholm, Sweden; Università degli Studi di Milano, ITALY

## Abstract

PCSK9-inhibitors (PCSK9i) are new drugs recently approved to lower LDL-cholesterol levels. However, due to the lack of long-term clinical data, the potential adverse effects of long-term use are still unknown. The *PCSK9* genetic locus has been recently implicated in mood disorders and hence we wanted to assess if the effect of PCSK9i that block the PCSK9 protein can lead to an increase in the incidence of mood disorders. We used genetically-reduced PCSK9 protein levels (pQTLs) in plasma, serum, cerebrospinal fluid as a proxy for the effect of PCSK9i. We performed Mendelian randomization analyses using PCSK9 levels as exposure and mood disorder traits major depressive disorder, mood instability, and neuroticism score as outcomes. We find no association of PCSK9 levels with mood disorder traits in serum, plasma, and cerebrospinal fluid. We can conclude that genetically proxied on-target effect of pharmacological PCSK9 inhibition is unlikely to contribute to mood disorders.

## Introduction

PCSK9 inhibitors (PCSK9i) are a new class of drugs prescribed to reduce uncontrolled levels of low-density lipoprotein cholesterol (LDL-C), especially where the gold standard treatment of statins and ezetimibe (NPC1L1 inhibitor) fails to control LDL-C levels [1]. As PCSK9i were only approved in 2015–16 [2], there is no long-term clinical data in which to assess possible adverse drug reactions (ADRs). As ADRS leads to increased morbidity and mortality and a higher burden on the healthcare system [3], it is important to explore these as early as possible. For statins, a drug that blocks HMGCR (3-hydroxy-3-methyl-glutaryl-coenzyme A reductase), it took several decades of use for enough evidence to be gathered to conclusively demonstrate an increased type 2 diabetes incidence with its long-term use [4].

In contrast, genetic analyses make it possible to predict ADRs in the absence of clinical data by using techniques such as Mendelian randomization (MR), which investigates causal relationships between exposures (proxied with genetic data) and outcomes [5, 6]. Genetic variants that reduce gene expression (eQTLs) or protein levels (pQTL) [7] can thus be used a proxy for a drug that reduces levels of, or blocks the function of a gene or its product.

Previously, we uncovered associations between genetic variants in the *PCSK9* locus and mood disorder traits [8]. Mood disorders include the traits mood swings or instability, neuroticism, anxiety, mania, and major depressive disorder (MDD). The global burden of such conditions is significant, with the global number of disability-adjusted life years (DALYS) attributed to it being 125.3 million as of 2019, contributing to 4.9% of the proportion of global DALYS [9]. If the usage of PCSK9i indeed increases to mood disorders, the burden to both the patients and health services will increase. Although PCSK9i do not cross the blood-brain barrier under normal conditions, various disease states can modify the permeability of the blood-brain barrier [10]. Hence, it is essential that this association be further explored.

PCSK9i work as either single interfering RNA (siRNA) to block mRNA and prevent its translation [11], or are monoclonal antibodies that bind to the protein and block the function of the protein itself [11]. Previously, we have demonstrated that *PCSK9* gene expression data showed no association with mood disorder traits [12], suggesting that siRNA PCSK9i usage are unlikely to lead to the mood disorder traits mood instability, neuroticism, or MDD. However, expanding the assessment to PCSK9i which directly target protein levels is needed to assess the possibility of ADRs from this alternative modality.

In this study, we used genetically predicted protein levels of PCSK9 to proxy the pharmacological effect of PCSK9i, and MR analyses to explore the possibility that protein-targeting PCSK9i might increase the risk of mood disorder traits in the long-term. We had used pQTL (genetic variants modulating PCSK9 protein levels) from serum, plasma, brain, and cerebrospinal fluid (CSF) to proxy the effect of PCSK9i. Despite PCSK9 being produced primarily in the liver, protein levels in the plasma and serum provide an overall effect of the protein level [13], whereas protein levels from brain and CSF can assess any localized effects.

## Results

### PCSK9 protein levels in plasma or serum are not associated with mood disorder traits

#### AGES cohort (serum)

**Fig 1:**
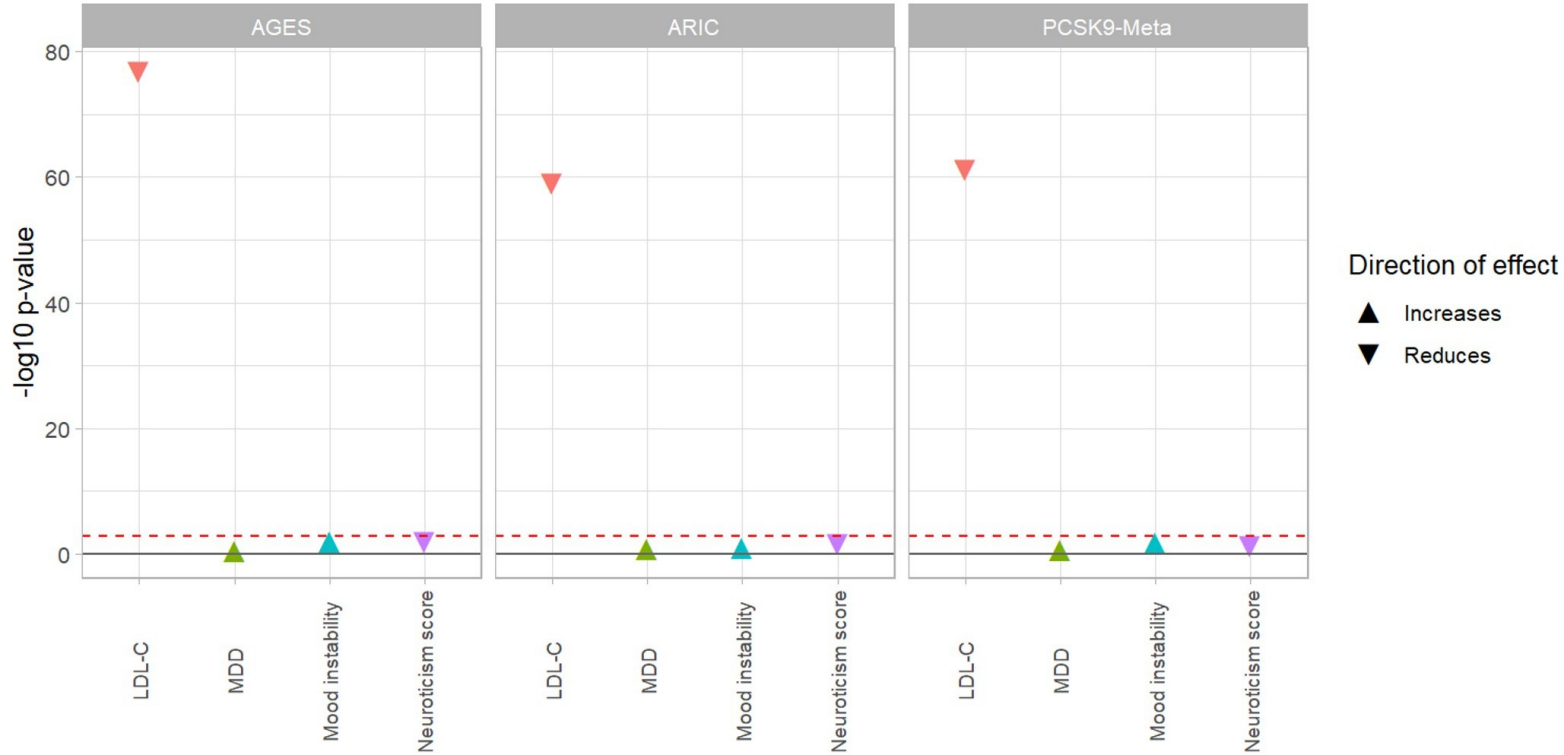
Association of reduced PCSK9 protein levels in the plasma and serum with mood disorder traits with stringent p-value threshold. SMR multi-SNP analyses was performed using cis-pQTLs as instruments to assess association of PCSK9 levels (exposure) with mood disorder traits (MDD, mood instability, and neuroticism score) as outcomes. On the X-axis are the outcomes (mood disorder traits and LDL-C as a positive control). The Y-axis depicts the -log10(p-value) for the SMR associations using multiple SNP. Three datasets were used for PCSK9 protein levels: ARIC (plasma), AGES (serum), and PCSK9 meta-GWAS (serum and plasma combined). The direction of the arrow represents the direction of effect with respect to reduction of PCSK9 levels–triangle pointing up: reduction of PCSK9 levels increases the outcome levels/risk, triangle pointing down: reduction of PCSK9 levels reduces the outcome levels/risk. The red line signifies the Bonferroni corrected p-value threshold of 0.0031.

In both relaxed and stringent threshold of pQTL, we found no association between genetically predicted reduction in PCSK9 levels and MDD (relaxed-P_SMR-multi_ = 0.410, stringent-P_SMR-multi_ = 0.290), or mood instability (relaxed-P_SMR-multi_ = 0.004, stringent-P_SMR-multi_ = 0.07) in either of the sets. However, a significant association was observed of reduced PCSK9 levels with reduction in neuroticism score in the relaxed set (relaxed-P_SMR-multi_ = 0.002) (data in S3 Table) but not the stringent set (stringent-P_SMR-multi_ = 0.010) (Fig 1, Table 1, data in S4 Table). There was a significant association between reduced PCSK9 levels in the serum and reduced LDL-C levels (positive control) in both the stringent (P_SMR-multi_ = 1.27x10^-77^) and relaxed (P_SMR-multi_ = 3.69x10^-68^) sets. The p-value for the HEIDI test in both the relaxed and stringent set for all mood disorder trait outcomes are above the P_HEIDI_≥0.01 threshold, indicating that the observed effect sizes are not due to heterogeneity.

**Table 1:** SMR results for PCSk9 protein levels as exposure and various outcomes.

Tissue	Cohort	Outcome	beta	SE	P value (SMR_multi_)
Serum	AGES	LDL-C	0.316919	0.024767	1.27E-77
Serum	AGES	MDD	-0.00374	0.002038	0.290747
Serum	AGES	Mood instability	-0.00221	0.00362	0.072199
Serum	AGES	Neuroticism score	0.040475	0.025794	0.010733
Plasma	ARIC	LDL-C	0.333598	0.023397	1.08E-59
Plasma	ARIC	MDD	-0.00394	0.002141	0.106571
Plasma	ARIC	Mood instability	-0.00232	0.00381	0.09074
Plasma	ARIC	Neuroticism score	0.042605	0.027112	0.0225
Plasma + Serum	PCSK9-Meta	LDL-C	1.09012	0.080079	6.3E-62
Plasma + Serum	PCSK9-Meta	MDD	-0.01286	0.007002	0.209872
Plasma + Serum	PCSK9-Meta	Mood instability	-0.00759	0.012452	0.01269
Plasma + Serum	PCSK9-Meta	Neuroticism score	0.139224	0.088647	0.039906
CSF	N/A	LDL-C	0.097492	0.025671	7.42E-16
CSF	N/A	MDD	0.010267	0.006895	0.600022
CSF	N/A	Mood instability	-0.00876	0.012325	0.014666
CSF	N/A	Neuroticism score	-0.11899	0.08768	0.010325

Instruments used were stringent (P_pQTL_≤5x10^-8^) pQTLs.

#### ARIC cohort (plasma)

We found no association between PCSK9 reduced levels, and MDD (relaxed-P_SMR_ = 0.190, stringent-P_SMR_ = 0.106) and mood instability (relaxed-P_SMR-multi_ = 0.012, stringent-P_SMR-multi_ = 0.090) in either the relaxed or stringent set. However, the association between reduced PCSK9 levels and increased neuroticism score (P_SMR-multi_ = 0.001) was significant in the relaxed set (data in S2 Table), but not in the stringent set (P_SMR-multi_ = 0.022) (Fig 1 and Table 1, data in S3 Table). The association of reduced PCSK9 levels in the plasma with reduced LDL-C levels was significant in both the stringent (P_SMR-multi_ = 1.07x10^-59^) and relaxed (P_SMR-multi_ = 1.58x10^-62^) set. The p-value for the HEIDI test in both the relaxed and stringent set for all mood disorder trait outcomes are above the P_HEIDI_≥0.01 threshold, indicating that the observed effect sizes are not due to heterogeneity.

#### PCSK9 meta-analyses cohort (serum + plasma)

There was no association between reduced PCSK9 protein levels and MDD (stringent-P_SMR-multi_ = 0.209, relaxed-P_SMR-multi_ = 0.253), mood instability (relaxed-P_SMR-multi_ = 0.019, stringent-P_SMR-multi_ = 0.012), or neuroticism score (relaxed-P_SMR-multi_ = 0.047, stringent-P_SMR-multi_ = 0.039) (Fig 1, Table 1, data in S2 and S4 Tables). Reduced PCSK9 levels were significantly associated with reduced LDL-C levels (relaxed-P_SMR-multi_ = 8.514x10^-60^, stringent-P_SMR-multi_ = 6.29x10^-62^) in both sets. The p-value for the HEIDI test in both the relaxed and stringent set for all mood disorder trait outcomes are above the P_HEIDI_≥0.01 threshold, indicating that the observed effect sizes are not due to heterogeneity.

### PCSK9 levels in the brain and CSF are not associated with mood disorder traits

**Fig 2:**
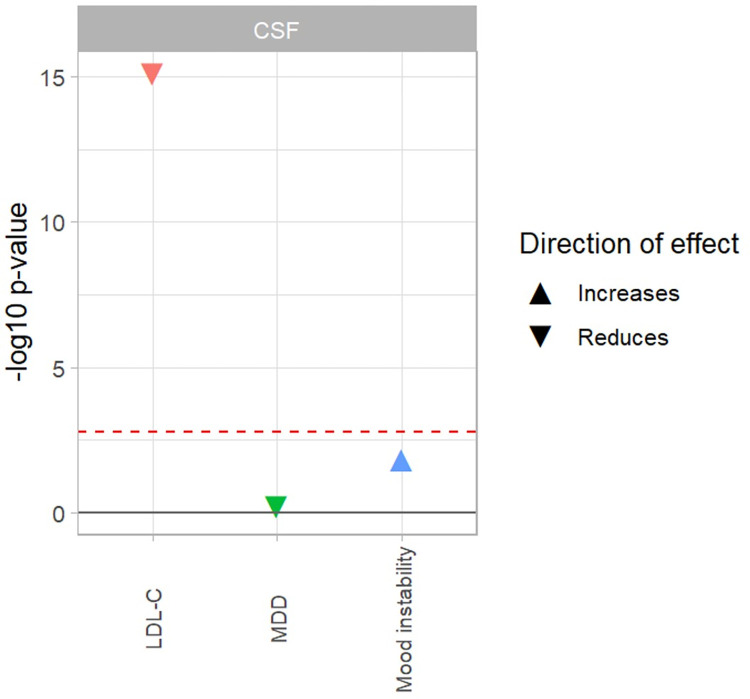
Association of reduced PCSK9 protein levels in the CSF with mood disorder traits. SMR multi-SNP analyses was performed using cis-pQTLs as instruments to assess association of PCSK9 levels (exposure) with mood disorder traits MDD, and mood instability as outcomes. The x-axis represents the three mood disorder traits and LDL-C as positive control. The y-axis depicts the -log10(p-value) for the SMR associations using multiple SNP. The shape represents the direction of effect with respect to reduction of PCSK9 levels: triangle pointing up: reduction of PCSK9 levels increases the outcome levels/risk, triangle pointing down: reduction of PCSK9 levels reduces the outcome levels/risk. Red line signifies the Bonferroni corrected p-value threshold of 0.0031.

We found that reduced CSF PCSK9 levels were not significantly associated with MDD (relaxed-P_SMR-multi_ = 0.816, stringent-P_SMR-multi_ = 0.600), and mood instability (relaxed-P_SMR-multi_ = 0.022, stringent-P_SMR-multi_ = 0.014) in either the relaxed or stringent set (Fig 2, Table 1, data in S3 Table). A significant association was found between reduced CSF PCSK9 levels and reduced LDL-C levels in both the relaxed (P_SMR-multi_ = 3.75x10^-18^) and stringent (P_SMR-multi_ = 7.41x10^-16^) set. However, the power of the MR analyses between PCSK9 levels in CSF and neuroticism score was very low at 0.41, and hence was not reported. The p-value for the HEIDI test in both the relaxed and stringent set for all mood disorder trait outcomes, except for mood instability, are above the P_HEIDI_≥0.01 threshold, indicating that the observed effect sizes are not due to heterogeneity.

### Concordance of results using traditional MR methods

We performed a traditional two sample MR IVW test to ascertain if the SMR results are concordant. We found that in the serum, plasma, and CSF i.e. all tissues tested, there PCSK9 protein levels were not significantly associated with the mood disorder traits of mood instability, neuroticism score, and MDD. LDL-C levels were used as a positive control and we found that genetically proxied reduction of PCSK9 protein levels were significantly associated with lower levels of LDL-C (Table 2).

**Table 2:** Associations between PCSK9 protein levels and various mood disorder traits using two sample MR (IVW) test.

Cohort	Outcome	Beta	SE	P	MREgger intercept	MREgger P	Cochrane Q	Cochrane Q P
AGES	MDD	-0.0016	0.0012	0.1700210	0.000266	0.660635	5.793494	0.760395
AGES	LDL-C	0.2301	0.0280	1.899E-16	-0.01119	0.449798	392.8453	4.6E-79
AGES	Mood swings	-0.0003	0.0035	0.93945	-0.00307	0.075537	25.08908	0.002874
AGES	Neuroticism score	0.0426	0.0192	0.0264594	-0.01883	0.036636	14.65366	0.100899
CSF	MDD	0.0092	0.0058	0.1145950	0.000228	0.848622	0.727283	0.69514
CSF	LDL-C	-0.0073	0.1832	0.968312	-0.04487	0.119959	252.5646	1.82E-54
CSF	Mood swings	-0.0082	0.0253	0.745302	0.00423	0.399427	19.21127	0.000247
CSF	Neuroticism score	-0.1312	0.1730	0.4480664	0.033222	0.309879	17.86788	0.000468
ARIC	MDD	0.0005	0.0031	0.8648145	N/A	N/A	0.036578	0.848326
ARIC	LDL-C	0.2635	0.0118	2.10E-110	N/A	N/A	1.103398	0.293522
ARIC	Mood swings	-0.0059	0.01150	0.6033390	N/A	N/A	4.177413	0.040966
ARIC	Neuroticism score	0.03049	0.07683	0.6914428	N/A	N/A	3.70458	0.054263
PCSK9 metaGWAS	MDD	0.0050	0.0046	0.2732174	-0.00071	0.122539	12.18164	0.273084
PCSK9 metaGWAS	LDL-C	-0.4629	0.3388	0.1718988	0.060804	0.068501	N/A	N/A
PCSK9 metaGWAS	Mood swings	0.0126	0.0114	0.2696879	0.00094	0.440231	23.90961	0.007844
PCSK9 metaGWAS	Neuroticism score	-0.0094	0.0884	0.9149049	0.011857	0.193204	28.42872	0.001541

Instruments used were stringent (P_pQTL_≤5x10^-8^) pQTLs. MR Egger intercept and Cochrane Q test assess the pleiotropy and heterogeneity respectively.

## Discussion

We specifically assessed whether long term PCSK9i use might increase the risk of mood disorder traits by using genetically reduced protein levels as a proxy for the effect of PCSK9i. We also investigated PCSK9 levels in CSF to reflect a more localized effect of PCSK9i.

We found no significant associations of reduced PCSK9 levels (serum and plasma) with increased mood disorder traits using the stringent set of pQTLS, suggesting that the variants strongly and specifically associated with PCSK9 protein levels do not influence neuroticism. The increased statistical power when using the relaxed set of pQTLS also comes with the trade-off of reduced specificity of the genetic instruments (i.e. these variants might be acting through other pathways in addition to the PCSK9 protein levels). Therefore, the associations of the ARIC and AGES relaxed set of pQTLS with (an effect on) neuroticism score, when viewed alongside the results of the stringent set of pQTLS is not convincing. That the same is not observed with the meta-analyses could be due to the larger sample size in the meta-analyses, which provides relaxed pQTL associations closer to the “true” effects (in this case closer to null) on PCSK9 protein levels and reduces the impact of study-specific outliers. Overall, we found that PCSK9 levels using pQTLs derived in the plasma, serum, and in combined plasma and serum tissues were not associated with mood-disorder traits MDD, mood instability, and neuroticism score.

This work follows on from a previous genetic study that explored the impact of *PCSK9* genetic variants on a variety of cardiometabolic and psychiatric-related phenotypes [8]. That study found associations with cardiovascular phenotypes, in line with known functions of *PCSK9* effects. The findings on mood traits, that could not be explained by current understanding of *PCSK9* functions, required further investigation, hence this study. A strength of our study is the very specific hypothesis tested, alongside “positive control” analyses of known effects. We cannot exclude the possibility of effects on other organs that were not explored.

Other strengths of this study include the concordance of our results across multiple tissues and cohorts and replicating the SMR analyses using a traditional MR method. Limitations of our study includes the lack of PCSK9 pQTLs in the brain, as no cis-pQTLs were detected for PCSK9 at either the relaxed or stringent p-value threshold. Hence, as PCSK9 is expressed in the brain, therefore it would be useful to investigate this further through PCSK9 pQTLs in the brain. Similarly, we were unable to analyse the impact of CSF PCSK9 levels and neuroticism score. Analyses are limited to European ancestry, however it is important to determine whether the effect is consistent across different ancestries. For future directions, we aim to also to incorporate pQTLs from different tissues if and when they become available.

The analyses were conducted in an effort expand on our previous work which studied the effect of targeting RNA as a mode of PCKS9 inhibition on mood-disorder traits. For this study, we focused on PCSK9 protein blocking instead by using PCSK9 pQTLs. The results for both our studies are consistent: long-term effect of PCSK9 inhibition, whether at the mRNA or the protein level, are unlikely to have a negative impact on mood-disorders. However, this is a genetically proxied study using MR that estimates the lifetime causal effect of an exposure (in this case PCSK9 inhibition) with an outcome (in this case mood disorder traits). It is not a clinical study, but a genetic one which was be used to infer potential ADRs that may arise due to long-term reduction in the levels of PCSK9.

In summary, here we have demonstrated that long-term use of PCSK9is, proxied genetically, which target PCSK9 protein levels are unlikely to increase the risk of mood disorder traits, consistent with our previous work using *PCSK9* gene expression to mimic siRNA PCSK9is.

## Methods

### Genetic data for exposure

Summary statistics for genetic variants associated with levels of PCSK9 *(*cis-pQTLs, ±1Mb flanking the gene) in European ancestry population were extracted from AGES (n = 5368, serum) [14], ARIC (n = 7,213, plasma) [15], a meta-analysis of PCSK9 levels (n = 12,721, plasma and serum combined) [13], and brain (n = 458) and CSF (n = 971) samples [16]. Characteristics of cohorts were presented in the data in S1 Table. Cis-pQTLs were filtered based on the p-value for association with PCSK9 protein levels at a threshold of P_pQTL_≤1x10^-5^ (relaxed set) and P_pQTL_≤5x10^-8^ (stringent set). We explored the relaxed threshold, as too few pQTLS would mean low statistical power. Increasing the number of pQTLs in the analyses by using the relaxed set enables verification that null associations are true null (rather than lack of power). Cis-pQTLs were used as the instrumental variables for the MR analyses.

### Genetic data for outcomes

The mood disorder traits assessed in this study were mood instability (an emotional state defined as rapid oscillations of intense affect leading to behavioural consequences [17]), neuroticism score (a score fir the trait of neuroticism characterised by an experience of negative effects, irritability, anxiety and other negative mood effects [18]), and MDD (major depressive disorder: a complex psychiatric condition with a clinical presentation of depressed mood, loss of interest, changes in appetite or weight, and increased likelihood of suicidal ideation [19]). Summary statistics were obtained from European ancestry genome-wide association studies of mood instability (n = 451,619), neuroticism score (n = 374,323), and MDD (n = 217,584) [20–22]. We also used circulating LDL-C levels as a positive control, as reduction in PCSK9 levels leads to a reduction in circulating levels of LDL-C [23]. Data was extracted from the IEU open GWAS project database using the ieugwasr R package version 0.1.5 [21].

### Power calculations

Power calculations were conducted using mRnd web version (accessed 15/06/2023) [24]. We used the observed estimate for the effect of *PCSK9* protein levels on neuroticism score, MDD, and LDL-C. Where these were not available, we used reported estimates of *PCSK9* genetic risk score on the above outcomes instead [25, 26]. No reported estimates were available for *PCSK9* protein levels on mood instability and hence we could not calculate the power for this outcome. We assumed the type-I error rate (α) as 0.05. The parameters used for the power calculations are available in the data in S2 Table.

### Statistical analyses

Causal associations between PCSK9i, proxied by reduced PCSK9 protein levels, and mood disorder traits was assessed using two-sample MR. Specifically, we used Summary-based MR (SMR) v1.3.1 [27], which is a modified two sample MR method that integrates QTL and GWAS summary statistics to assess if the intermediate phenotype modulated by the QTLs is causally associated with the trait. We also performed the Heterogeneity in dependent instruments (HEIDI) test to test the null hypothesis that the SMR estimate is due to directional pleiotropy instead of true causality, rejecting the hypothesis with a P_HEIDI_<0.01.

The parameters used for SMR analyses were: P_pQTL_≤5x10^-8^ (stringent set) and P_pQTL_≤1x10^-5^ (relaxed set), linkage disequilibrium (LD) pruning threshold of r^2^<0.2, and exclusion of pQTLs in very high LD (r^2^>0.9) or minimal LD (r^2^<0.05) with the lowest P-value pQTL for the HEIDI test. The analyses and results from the brain tissues were excluded due to lack of power. The significance threshold was corrected for multiple testing and set at P-value <0.0031 (Bonferroni corrected for 4 outcomes in 4 cohorts). As the effects of pQTLs are very small, only the significance, not the effect size of the association was used to infer causality in this study.

We also repeated the analyses using the traditional MR methods to compare the SMR results. Inverse variance weighted (IVW) was used as the main MR method with sensitivity analyses: weighted median MR, MR Egger to assess pleiotropy and Cochrane Q test to assess heterogeneity. Instruments for the exposure, PCSK9 protein levels, used were cis-pQTLs from three different cohorts: ARIC, AGES, and PCSK9-Meta GWAS with P_pQTL_≤5x10^-8^. These instruments were clumped at an LD threshold of r^2^<0.2 using the European superpopulation 1000Genomes Reference panel. The exposure summary statistics were then harmonised against the outcomes, mood instability, neuroticism score, MDD, and LDL-C levels, and then used to conduct the MR analyses. TwoSampleMR R package [28] was used to conduct the analyses.

No ethical approval was required as all data used in the study are publicly available and anonymised.

## Supporting information

S1 Table Cohort descriptions. (XLSX)

S2 Table Power calculations. (XLSX)

S3 Table SMR results for eQTLs with relaxed p-value threshold of p< = 1x10e-5. (XLSX)

S4 Table SMR results for eQTLs with relaxed p-value threshold of p< = 5x10e-8. (XLSX)
